# Supplementary material for: Molecular pathways behind acquired obesity: Adipose tissue and skeletal muscle multiomics in monozygotic twin pairs discordant for BMI
Source: Cell Rep Med. 2021 Mar 30;2(4):100226. doi: 10.1016/j.xcrm.2021.100226 (PMC8080113; doi:10.1016/j.xcrm.2021.100226)
Supplement: Document S1. Figure S1 [file mmc1.pdf]

Cell Reports Medicine, Volume *2*

Supplemental information

Molecular pathways behind acquired obesity: Adipose tissue and skeletal muscle multiomics in monozygotic twin pairs discordant for BMI

Birgitta W. van der Kolk, Sina Saari, Alen Lovric, Muhammad Arif, Marcus Alvarez, Arthur Ko, Zong Miao, Navid Sahebekhtiari, Maheswary Muniandy, Sini Heinonen, Ali Oghabian, Riikka Jokinen, Sakari Jukarainen, Antti Hakkarainen, Jesper Lundbom, Juho Kuula, Per-Henrik Groop, Taru Tukiainen, Nina Lundbom, Aila Rissanen, Jaakko Kaprio, Evan G. Williams, Nicola Zamboni, Adil Mardinoglu, Päivi Pajukanta, and Kirsi H. Pietiläinen

Supplemental Information

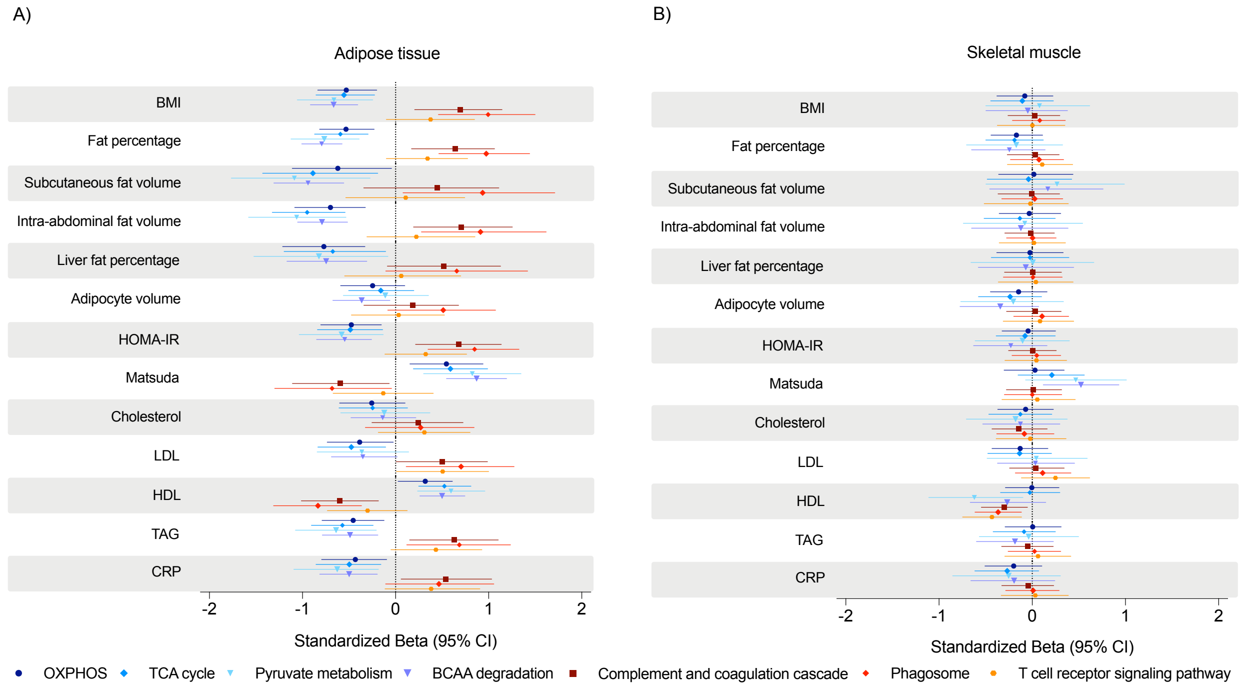

Supplemental Figure 1. Associations of four mitochondrial and three inflammatory pathways with clinical outcome measures. Related to figure 5. A) Standardized coefficients (βs) in linear mixed models with the clinical outcome among co-twins as the dependent variable, the scores of mitochondrial and inflammatory pathways as the fixed effect and family ID as the random effect, adjusted for sex, age and diabetes status in adipose tissue. B) Standardized coefficients (βs) in linear mixed models with the clinical outcomes among co-twins as the dependent variable, the scores of mitochondrial and inflammatory pathways as the fixed effect and family ID as the random effect, adjusted for sex, age and diabetes status in skeletal muscle. Error bars denote the 95% confidence intervals. BMI, body mass index; HDL, high-density lipoprotein; LDL, low-density lipoprotein; HOMA-IR, homeostatic model for the assessment of insulin resistance; CRP, C-reactive protein. Related to Figure 5.
